# Unraveling the history of limb bones

**DOI:** 10.7554/eLife.66506

**Published:** 2021-03-02

**Authors:** Holly N Woodward

**Affiliations:** Department of Anatomy and Cell Biology, Oklahoma State University Center for Health SciencesTulsaUnited States

**Keywords:** evolution, tetrapod terrestrialisation, haematopoiesis, stem amniotes, amphibians, propagation phase-contrast synchrotron microtomography, Other

## Abstract

Ancient fossils give clues as to when features of modern tetrapod bones emerged.

**Related research article** Estefa J, Tafforeau P, Clement AM, Klembara J, Niedźwiedzki G, Berruyer C, Sanchez S. 2021. New light shed on the early evolution of limb-bone growth plate and bone marrow. *eLife* **10**:e51581. doi: 10.7554/eLife.51581

Any land creature with a backbone and four limbs is related to a fish that started to crawl over 360 million years ago (Niedźwiedzki et al., 2010). Since then, evolutionary processes have shaped this ancestor into a brethren of four-legged ‘tetrapods’, from frogs to lizards to your pet dog. The fossil record, and in particular limb bones, provide scant but tantalizing clues about the stepwise changes that helped the early descendants of this fish to acquire the traits which allowed them to become fully terrestrial 300 million years ago.

Limbs first evolved as a way to adapt to life in shallow waters, but they became a game changer for land travel (Ahlberg, 2018). Over time, they acquired characteristic features; for instance, in modern tetrapods, limb growth generally takes place in the metaphysis – the ‘neck’ area near the end of long bones, which hosts a mineralized region known as the growth plate. There, cartilage cells organize into calcified columns, forming a characteristic three-dimensional fan-like meshwork (Hall, 2005). Today, limb bones also serve additional roles. While fish create red blood cells in the liver and kidney for example, most current species of tetrapods carry out this process in the marrow of their long bones (Akiyoshi and Inoue, 2012). Now, in eLife, Sophie Sanchez and colleagues based at Uppsala University, the European Synchrotron Radiation Facility, Flinders University and Comenius University – including Jordi Estefa (Uppsala) as first author – report new insights into when these characteristics of limb bones emerged in tetrapods during the water-to-land transition (Estefa et al., 2021).

To explore how limb bones developed in early tetrapods, the team harnessed synchrotron micro-computed tomography, a technique that uses a high-powered particle beam scanner to virtually ‘slice’ up and image thin layers of fossilized bone. The resulting images are then stacked together using computer processing to produce a detailed three-dimensional model of the internal structure of the limb bone.

The analyses revealed that the fan-like structures that form the growth plate in the metaphysis were present both in the ancient amphibian *Metoposaurus* – which mainly lived in water – and two ‘amniote’ species, *Seymouria* and *Discosauriscus*, which could reproduce on land. This suggests that growing bone by calcifying cartilage columns is a process that appeared in earlier, water-bound tetrapods, and has a shared origin between amphibians and amniotes. The way that tetrapod limbs grow today was therefore already present in our earliest four-limbed ancestors, long before the transition to land (Figure 1).

**Figure 1.**
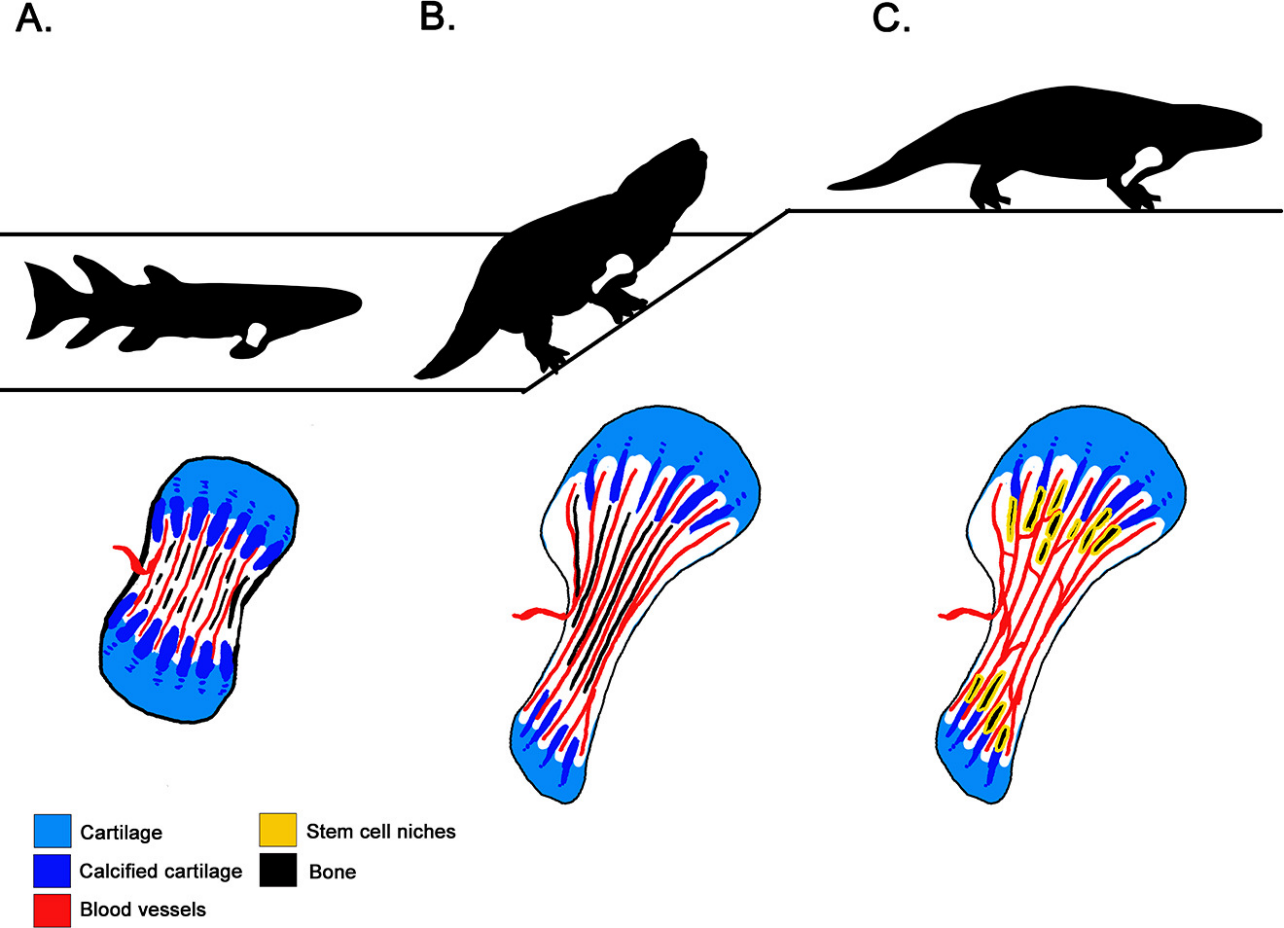
How tetrapods acquired new bone characteristics as they transitioned from water to land. (**A**) About 380 million years ago, lobe-finned tetrapods were still water-bound (top). Yet, lengthwise cross sections of their forelimb bones (bottom) show that they had already evolved limbs that elongate through calcified cartilage columns (dark blue) within the metaphysis – the area near the extremities of the bones that features a ‘growth plate’ formed of cartilage (light blue). Marrow processes — the blood vessels (red) between the mineralized columns in the growth plate — were also present at this stage. However they did not communicate freely with the open cavity inside the shaft. (**B**) Tetrapods that first ventured onto land 360 million years ago (top) also elongated their limbs at the growth plate. Their bones do show evidence of marrow processes occurring within the metaphysis (bottom), but they still produce red blood cells via their liver and kidney. Indeed, a trait necessary for red blood cell production in the bone is missing: the blood vessels of the marrow processes open into small connected cavities in the bone rather than communicating with the open marrow cavity. (**C**) Fully terrestrial tetrapods appeared 300 million years ago (top), and they retained the fan-like growth plate of their ancestors (bottom). However, the cavities within their bones indicate that the marrow processes were interconnected via blood vessels, and that they communicated with the bone marrow. This suggests that red blood cells were now produced within bone.

In most current tetrapods, the columns in the metaphysis host stem cell niches that produce the precursor cells which mature into red blood cells (Orkin and Zon, 2008). For this arrangement to work, the niches need to be connected to the primary blood vessels that invade the marrow cavity: this allows red blood cells to be released from the bone into the systemic circulation (Calvi et al., 2003; Zhang et al., 2003; Tanaka, 1976). However, Estefa et al. found that in older, water-bound tetrapods, the spaces within columnar meshwork did not communicate with the bone marrow cavity. In fact, the earliest evidence of communication between these two structures was found in fully terrestrial tetrapods that could reproduce on land 300 million years ago. Crucially there was no evidence of this connection in tetrapods from 360 million years ago, even though these creatures could already explore land (Figure 1). Producing red blood cells inside the bone marrow was thought to be required for life out of water (e.g., Kapp et al., 2018), but these results indicate that this may not be the case. Instead, they suggest that bone marrow and red cell production appeared successively rather than simultaneously during evolution, even though these characteristics are intimately linked in tetrapods today.

Next, the team endeavors to discover exactly at what point the site of red blood cell production migrated to bone marrow – and why. If this event took place in the first tetrapods to explore land, then all their descendants could have inherited this trait. If the migration happened later, when terrestrial tetrapods had already started to occupy distinct habitats, then red blood cell production in bone marrow may have evolved several times independently. Finally, pinning down when or in which taxon red blood cell production first relocated to the marrow will help to understand the environmental or biological factors that triggered this migration. In turn, this could shed light on the subsequent biological innovations that became unlocked when red blood cells started to be produced inside bones.
